# Low Prevalence of *NOTCH2NLC* GGC Repeat Expansion in White Patients with Movement Disorders

**DOI:** 10.1002/mds.28302

**Published:** 2020-10-07

**Authors:** Wai Yan Yau, Jana Vandrovcova, Roisin Sullivan, Zhongbo Chen, Anna Zecchinelli, Roberto Cilia, Stefano Duga, Malgorzata Murray, Susana Carmona, Genomics England Research Consortium, Viorica Chelban, Hiroyuki Ishiura, Shoji Tsuji, Zane Jaunmuktane, Chris Turner, Nicholas W. Wood, Henry Houlden

**Affiliations:** ^1^ Department of Neuromuscular Diseases, UCL Queen Square Institute of Neurology University College London London United Kingdom; ^2^ Department of Neurodegenerative Diseases, UCL Queen Square Institute of Neurology University College London London United Kingdom; ^3^ Biobank Centro Parkinson e Parkinsonismi ASST Pini CTO Milan Italy; ^4^ Fondazione IRCCS Istituto Neurologico Carlo Besta, Parkinson and Movement Disorders Unit Milan Italy; ^5^ Department of Biomedical Sciences Humanitas University Milan Italy; ^6^ IRCCS Istituto Clinico Humanitas, Rozzano Milan Italy; ^7^ UK Dementia Research Institute (UK DRI) at UCL London United Kingdom; ^8^ Genomics England London UK; ^9^ Department of Neurology The University of Tokyo Tokyo Japan; ^10^ Department of Molecular Neurology The University of Tokyo Tokyo Japan; ^11^ Institute of Medical Genomics International University of Health and Welfare Chiba Japan; ^12^ Divison of Neuropathology National Hospital for Neurology and Neurosurgery London United Kingdom; ^13^ Department of Clinical and Movement Neurosciences UCL Queen Square Institute of Neurology London United Kingdom; ^14^ MRC Centre for Neuromuscular Diseases National Hospital for Neurology and Neurosurgery London United Kingdom; ^15^ Neurogenetics Unit National Hospital for Neurology and Neurosurgery London United Kingdom

**Keywords:** trinucleotide repeat diseases, spinocerebellar ataxia, Parkinson's disease, tremor, multiple system atrophy

## Abstract

**Background:**

The objective of this study was to determine the prevalence of the GGC‐repeat expansion in *NOTCH2NLC* in whites presenting with movement disorders.

**Methods:**

We searched for the GGC‐repeat expansion in *NOTCH2NLC* using repeat‐primed polymerase chain reaction in 203 patients with essential tremor, 825 patients with PD, 194 patients with spinocerebellar ataxia, 207 patients with “possible” or “probable” MSA, and 336 patients with pathologically confirmed MSA. We also screened 30,008 patients enrolled in the 100,000 Genomes Project for the same mutation using ExpansionHunter, followed by repeat‐primed polymerase chain reaction. All possible expansions were confirmed by Southern blotting and/or long‐read sequencing.

**Results:**

We identified 1 patient who carried the *NOTCH2NLC* mutation in the essential tremor cohort, and 1 patient presenting with recurrent encephalopathy and postural tremor/parkinsonism in the 100,000 Genomes Project.

**Conclusions:**

GGC‐repeat expansion in *NOTCH2NLC* is rare in whites presenting with movement disorders. In addition, existing whole‐genome sequencing data are useful in case ascertainment. © 2020 The Authors. *Movement Disorders* published by Wiley Periodicals LLC on behalf of International Parkinson and Movement Disorder Society

Adult‐onset neuronal intranuclear inclusion disease (NIID) is a progressive neurodegenerative disease with clinical manifestations such as dementia, myopathy, leukoencephalopathy, autonomic neuropathy, peripheral neuropathy, sporadic encephalitic episodes, and a mixture of movement disorders.^1^ Previously, clinicians relied on ancillary tests to make a diagnosis of NIID: characteristic MRI head features of high signal intensity in the corticomedullary junction in diffusion‐weighted image^1^ and skin biopsy showing pathological hallmarks of eosinophilic ubiquitin‐positive and p62‐positive intranuclear inclusions in adipocytes, fibroblasts, and sweat glands.^2^

The major breakthrough occurred with the discovery of GGC‐repeat expansion in *NOTCH2NLC* as the causative mutation for NIID.^3^, ^4^ Intriguingly, the same mutation was found in 11 of 197 (5.5%), 3 of 205 (1.5%), and 5 of 189 (2.6%) Chinese families with essential tremor (ET), Parkinson's disease (PD), and multiple system atrophy (MSA), respectively.^5^, ^6^, ^7^ These findings suggest that this repeat expansion may be involved in the pathogenesis of ET, PD, and MSA in the East Asian population. However, the role of this mutation in other ethnic groups is currently unknown. We aimed to establish the prevalence of GGC‐repeat expansion in *NOTCH2NLC* in white patients with movement disorders.

## 1 Methods

### 1.1 Participants

We analyzed white patients with movement disorders: 203 with ET, of whom 57 probands (28%) have a positive family history; 825 with PD, of whom 274 are young onset (age at onset, ≤50 years); 194 with spinocerebellar ataxia (SCA); 207 with “possible” or “probable” MSA; and 336 with pathologically confirmed MSA. SCA and clinical MSA patients were enrolled from the National Hospital for Neurology and Neurosurgery (NHNN), UK. These patients tested negative for the common repeat expansion ataxia genes *SCA* 1–3, 6, 7, 12, and 17 and Friedreich's ataxia. We received genomic DNA (gDNA) of all PD patients from the NINDS Human Genetics and Cell Line Repository, United States, and gDNA of all ET patients from the Parkinson Institute Biobank, ASST G. Pini‐CTO, Milan, Italy. We obtained extracted gDNA of the pathologically confirmed MSA from brain banks around the world. The diagnoses of ET, MSA, and PD were achieved using established criteria.^8^, ^9^, ^10^ In addition, we analyzed 30,008 patients in the 100,000 Genomes Project, which performed whole‐genome swequencing (WGS) in individuals with undiagnosed rare disorders.^11^ This cohort contained patients with neurological and nonneurological phenotypes who were of White, Asian, and Black ethnic background. Of these, 383 white British probands had early‐onset familial Parkinson's disease. The joint ethics committee of UCL Queen Square Institute of Neurology, and NHNN approved this study (UCLH: 04/N034).

### 1.2 Repeat‐Primed Polymerase Chain Reaction and Southern Blot Analysis

To test for the presence of GGC‐repeat expansion at *NOTCH2NLC*, we performed repeat‐primed polymerase chain reaction (RP‐PCR) using primers and protocol previously published^3^ (Table S1), followed by fragment‐length analysis on an ABI 3730xl DNA analyzer with 500Liz ladder and GeneMapper software (v5). Expansions with a characteristic sawtooth pattern were identified and put forward for Southern blotting. The Southern blotting (SB) protocol for *NOTCH2NLC* was performed as outlined by Ishiura et al.^3^ gDNA (5 μg) was digested with SacI. We subcloned plasmids of target genetic fragment (pTA2; Toyobo) and generated DIG‐labeled PCR probes 1 and 2 for hybridization (Table S1). The probes were hybridized overnight at 48°C and the membrane washed with 0.1× saline sodium citrate and 0.1% sodium dodecyl sulfate at 68°C twice for 15 minutes each.

### 1.3 Short‐Read Whole‐Genome Sequencing Analysis and Long‐Read Sequencing

Short‐read WGS was performed as a part of the 100,000 Genomes Project.^11^ All samples analysed in the study were aligned to the genomic build GRCh38. ExpansionHunter v3.0.0 and its supplied variant catalogue were used to estimate GGC‐repeat allele length within the *NOTCH2NLC* region.^12^ ExpansionHunter is the default tool in the 100,000 Genomes Project for detecting pathogenic repeat expansions; it has better accuracy in identifying expanded repeats and estimating their allele sizes than comparative bioinformatic tools.^12^, ^13^ To estimate background frequencies of the full repeat (GGC)n(GGA)n(GGC)n, we also performed an ExpansionHunter analysis using a custom repeat definition. Samples with allele length in the top 0.01 percentile were further visualized and manually inspected using GraphAlignmentViewer.

Library preparation of nanopore sequencing was performed with 3 μg of gDNA and a Genomic DNA by Ligation kit (SQK‐LSK109), followed by sequencing on the PromethION sequencer using Flo‐PRO002 flow cells and Guppy high‐accuracy base calling (Oxford Nanopore Technologies). Reads were aligned to the GRCh38 reference using minimap2 and the target region was analyzed using RepeatHMM and Integrative Genomics Viewer.^14^, ^15^, ^16^

### 1.4 Skin Biopsy and Pathology

Skin with a diameter of 5 mm was taken from the patient's medial forearm, and specimens were fixed in 10% buffered formaldehyde followed by processing for paraffin histology. Pathological examinations included hematoxylin and eosin staining and immunostaining with anti‐p62 antibody (1:100; BD Transduction) and antiubiquitin antibody (1:1200; Santa Cruz) using Ventana immunostainers as per the manufacturer's guidelines. Then 3,3‐diaminobenzidine was used as chromogen, and negative and positive controls were immunostained in parallel.

## 2 Results

### 2.1 Mutation Screening

**FIG. 1.**
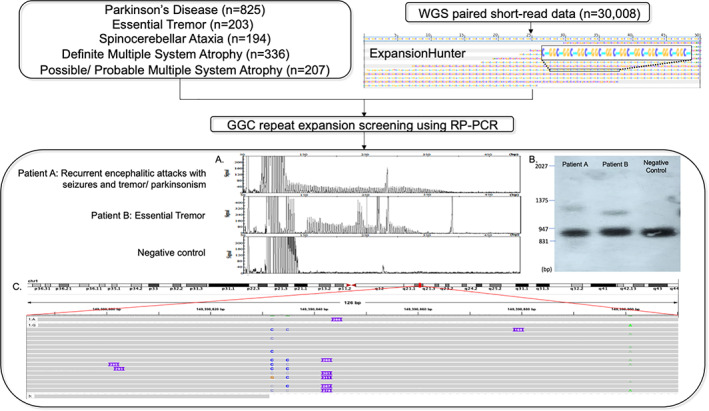
Flowchart of GGC‐repeat expansion screening in whites. (**A**) RP‐PCR of patient A, patient B, and negative control. (**B**) Southern blot of patients A and B. (**C**) Nanopore sequencing of patient A in the 5′ region of *NOTCH2NLC* on IGV.

A flowchart of the mutation screening and its results is shown in Figure 1. RP‐PCR screening of GGC‐repeat expansion in *NOTCH2NLC* identified 1 patient in the ET cohort (patient B) but none in the PD, SCA, pathologically confirmed MSA, and clinical MSA cohorts. We manually visualized the ExpansionHunter analysis of WGS data in patients with ≥58 repeats (top 0.01%) for which DNA was available for RP‐PCR: only 1 patient (patient A) carried the expansion. The false‐positive cases showed interrupted GGC‐repeat tracts and low number of reads, in contrast with patient A, who had multiple reads of pure GGC‐repeat tracts of various lengths, supporting the expanded allele (Fig. S1). Repeat expansions for both patients were confirmed by SB and/or long‐read sequencing: the GGC‐repeat size in patient A was estimated to be 118 on SB and 92–106 on nanopore sequencing, whereas the repeat size in patient B was estimated to be 90 on SB (Fig. 1B,C, Figs. S2 and S3). Patient A had a pure (GGC)_n_ expansion without GGA interruption.

### 2.2 Comparison of GGC Repeat Length and Motif in Different Ethnic Populations

**FIG. 2.**
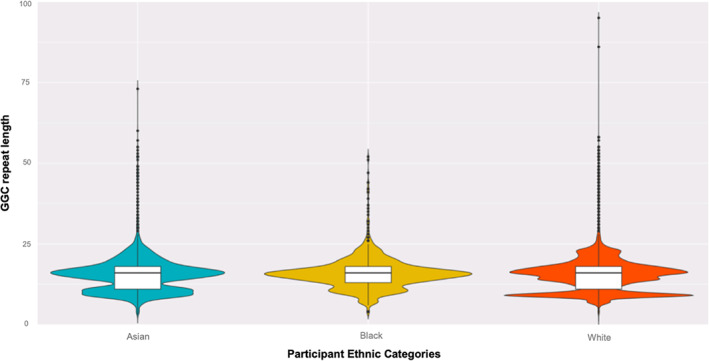
Violin plot of 30,008 participants enrolled in 100,000 Genomes Project: allelic distribution of the GGC repeat is similar across different ethnic groups. Box indicates the interquartile range, horizontal line within the box the median, and the whiskers the 95% confidence intervals.

WGS‐based analysis using ExpansionHunter did not identify population‐related differences in allele length (Fig. 2). The most common repeat in all populations was (GGC)_n_(GGA)_2_(GGC), followed by (GGC)_n_ (GGA)_3_(GGC), which is consistent with the findings in Japanese control subjects.^3^

### 2.3 Case Studies

Patient A is a 60‐year‐old Ukrainian woman with recurrent encephalopathy, migraine, and reversible focal neurological deficits. Her major encephalopathic episodes occurred at age 39, 49, and 56 years, complicated by status epilepticus. CSF was bland, with elevated protein, and MRI head showed multifocal cortical and subcortical signal abnormalities without hyperintensity in the corticomedullary junction in DWI sequences (Fig. S4A–H). Examination at age 60 years revealed dysarthria, hypomimia, bradykinesia, hypokinesia, resting and postural tremor of the upper limbs, global areflexia, and reduced pinprick and vibration in the distal lower limbs (video S1). DaTscan showed normal uptake of striatal dopamine transporter (Fig. S4I). Skin biopsy showed p62‐ and ubiquitin‐positive inclusions in serous glands, fibroblasts, and endothelium (Fig. S4J). Figures S5 and S6 provide an in‐depth case review and family tree of patient A.

Patient B was a 69‐year‐old Italian man who developed postural tremor of his arms at age 54 years. His family history was notable for both his mother and sister with late‐onset tremor‐dominant PD (Fig. S7). His clinical examination revealed isolated bilateral upper limb postural tremor without any sign of parkinsonism or other relevant signs of neurological involvement. The neuropsychometric tests did not reveal cognitive deficit. MRI of the head at age 54 years showed minimal white‐matter lesions at the corona radiata on T2‐weighted sequences, whereas DaTscan was normal.

## 3 Discussion

We report 2 patients carrying the GGC‐repeat expansion in *NOTCH2NLC*: 1 patient presenting with autosomal‐dominant ET and the other patient presenting with recurrent encephalopathy and postural tremor/ parkinsonism mimicking mitochondrial disorders. The prevalence of this expansion is 0.5% in our white ET cohort. Previous postmortem pathological case series supports the low frequency of this mutation in whites: intranuclear inclusions were found in fewer than 1% of brains in ET patients in whom the *FMR1* gene premutation was excluded.^17^ However, it remains uncertain whether GGC‐repeat expansion in *NOTCH2NLC* is a genetic cause of ET or a subtype in the phenotypic spectrum of NIID. A limitation of our study was our inability to obtain a skin sample and MRI head images of patient B, which may provide pathological and radiological evidence to support the diagnosis of NIID. Recent studies described 3 East Asian ET patients carrying this repeat expansion and intranuclear inclusions in skin biopsy: 2 of them manifested classical clinical and radiological features of NIID 10 years after initial tremor onset, but 1 patient continued to display pure ET phenotype after 4 decades of follow‐up.^18^, ^19^ These studies illustrate the variable clinical expressivity of NIID, which may be caused by genetic factors such as GGC‐repeat length, interruptions in the repeat tracts, or other unknown modifiers. Thereby, it is plausible that our patient displayed an ET phenocopy of NIID.

In addition, we successfully detected the pathogenic expansion in *NOTCH2NLC* in a patient using the computational tool ExpansionHunter, corroborating the utility of WGS data for screening repeat expansion disorders.^20^ We leveraged the WGS data in a large population cohort to demonstrate that the background repeat structure and allelic frequency of the GGC repeat in *NOTCH2NLC* between Whites and East Asians are comparable and do not explain the ethnic difference in NIID prevalence. The difference in prevalence may be a result of a founder effect in East Asian populations. Moreover, the absence of the GGC‐repeat expansion in the pathologically confirmed MSA cohort verifies that this expansion is not involved in the pathogenesis of MSA.

This study confirms the low frequency of GGC‐repeat expansion in *NOTCH2NLC* in whites with movement disorders. Clinicians should reserve testing of this mutation in white patients with classical symptoms of NIID but retained a high level of clinical suspicions in familial ET patients.

## Authors' Roles

(1) Research project: A. Conception, B. Organization, C. Execution; (2) Statistical Analysis: A. Design, B. Execution, C. Review and Critique; (3) Manuscript: A. Writing of the first draft, B. Review and Critique.

W.Y.Y.: 1A, 1B, 1C, 2A, 2B, 2C, 3A, 3B.

J.V.: 1A, 1B, 1C, 3B.

R.S.: 1C, 3B.

V.C.: 1C, 3B.

Z.H.: 1B, 3B.

M.M.: 1C, 3B.

S.C.: 1C, 3B.

H.I.: 1B, 3B.

S.T.: 1B, 3B.

A.Z.: 1B, 3B.

R.C.: 1B, 3B.

D.S.: 1B, 3B.

Z.J.: 1C, 3B.

C.T.: 1B, 3B.

N.W.W.: 1B, 3B.

H.H.: 1A, 1B, 3B.

## Financial Disclosures

None.

**Funding**: W.Y.Y. receives a PhD studentship from Ataxia UK and Rosetrees Trust and funding from a Nicholas Blair MDSANZ travelling fellowship. R.S. receives funding from MRC. Z.C. is supported by a clinical fellowship from the Leonard Wolfson Foundation. Z.J. is supported by the Department of Health's NIHR Biomedical Research Centre's funding scheme. SD is supported by PRIN (Programmi di Ricerca Scientifica di Rilevante Interesse Nazionale, Grant n. 2017228L3J, Program coordinator SD). CT is supported by the Department of Health's NIHR Biomedical Research Centre's funding scheme. This research was supported by Medical Research Council (MRC), Wellcome Trust Synaptopathies award, MRC Centre grant (G0601943), Ataxia UK, Rosetrees Trust, Brain Research UK, UCL ODA/LMIC award, MSA Trust, MDUK, Muscular Dystrophy Association (MDA), and UCL/UCLH National Institute for Health Research University College London Hospitals Biomedical Research Centre.

## Supporting information

**Video S1.** The neurological examination of patient A showed resting and postural tremor with bradykinesia.

**Table S1.** Primer sequences and thermocycling conditions
**Figure S1**. A visual representation of ExpansionHunter output generated by GraphAlignmentViewer for the proband captured multiple reads of pure GGC‐repeat tracts of various lengths (A) compared with the output of the false‐positive cases showing interrupted GGC‐repeat tracts and a smaller number of reads supporting the expanded allele (B).
**Figure S2**. Southern blot uncropped image.
**Figure S3**. Integrative Genomics Viewer of Oxford Nanopore Technologies long‐read sequencing uncropped image.
**Figure S4**. MRI head, DaTscan, and skin histopathology images of patient A.
MRI head of patient A at age 49 years during an episode of encephalopathy showed hyperintensities in the right temporoparietal cortical and subcortical regions and corpus callosum in T2 FLAIR sequences (A–C); routine scan with patient at age 60 years showed hyperintensities in left frontal gyrus and middle cerebellar peduncles in T2 FLAIR coronal sequences (D–E) and mild global cerebral atrophy in T1 sagittal sequence (F) but no evidence of nigrostriatal degeneration on T2‐weighted axial sequences or hyperintensity in the corticomedullary junction in DWI sequences (G–H). DaTscan showed normal uptake of striatal dopamine transporter (I). Skin biopsy of patient A showed p62‐positive intranuclear inclusions in endothelium (blue arrow), fibroblasts (red arrow), and serous glands (pink arrow). Upper row demonstrates p62 immunostaining with hematoxylin counterstain when viewed under a bright‐field microscope, and the bottom row shows the negatives of the bright‐field images. Scale bar: 10 μm (J).
**Figure S5**. In‐depth case review of patient A.
**Figure S6**. Four‐generation family tree of patient A. The number below an individual indicates the age in years.
**Figure S7**. Four‐generation family tree of patient B. The number below ane individual indicates the age in years.

**Appendix S1**. Members of Genomics England Research Consortium

## Data Availability

We will share anonymized data pertaining to this study with any qualified investigator.
