# Supplementary material for: Low Prevalence of NOTCH2NLC GGC Repeat Expansion in White Patients with Movement Disorders
Source: Mov Disord. 2020 Oct 7;36(1):251–5. doi: 10.1002/mds.28302 (PMC8436747; doi:10.1002/mds.28302)

Supplementary Table 1. Primers sequences and thermocycling conditions

	Primers	PCR	Cycling	Ramp Rate
RP-PCR	NBF19-R: 5'-AGCGCCCACAGCAGAGCGGC-3'<br>pGEX3'-(CGG)$_5$: 5'-CCGGGAGCTGCATGTGTCAGAGG(CGG)$_5$-3'<br>Fam-pGEX3': 5-FAM-CCGGGAGCTGCATGTGTCAGAGG-3'	Takara LA taq 2.5units<br>2X GC buffer II 25µl<br>dCTP/dTTP/dATP (2.5mM) 4µl<br>7 deaza-dGTP (2.5mM) 4µl<br>Primers: 180nmol/µl for NBPF19-R and fam-pGEX3'; 9nmol/µl for pGEX3'-(CGG)$_5$<br>gDNA 50ng<br>H_2O to 25µl	95°C 5mins<br>[95°C 30s,<br>98°C 10s,<br>62°C 30s,<br>72°C 2mins] X 50<br>4°C	$2.5°Cs^{-1}$<br>$2.5°Cs^{-1}$<br><br>$-1.5°Cs^{-1}$<br>$2.5°Cs^{-1}$
Plasmid Template	NBPF19_1: 5'-ACCGAGAAGATGCCCGCCCTGC-3'<br>NBPF19_4: 5'-CGCGCCTCGGAAAGAATAACAG-3'	TaKaRa LA Taq 2.5units<br>2XGC Buffer II 25µl<br>dNTP (2.5nM) 4µl<br>Primers: 180nmol/µl for NBF19_1 and NBF19_4<br>gDNA 50ng<br>H_2O to 25µl	98°C 1min<br>[98°C 10s,<br>57°C 30s,<br>68°C 30s] X 35<br>4°C	N/A

Probe 1	NBPF19_1: 5'-ACCGAGAAGATGCCCGCCCTGC-3'<br>NBPF19_1R: 5'-AACTGCCCACCTCCCTGCACC-3'	PCR DIG Probe Synthesis Kit (Roche):<br>Enzyme mix 0.75 µl<br>PCR DIG Probe synthesis mix 5 µl<br>10X PCR buffer with $MgCl_2$ 5µl<br>Primers: 180nmol/µl for NBF19_1 and NBF19_1R<br>gDNA 50ng<br>H_2O to 50µl	95°C 2mins<br>[95°C 30s,<br>55°C 30s,<br>72°C 45s] X 35<br>72°C 7mins<br>4°C	N/A
Probe 2	NBPF19_4F: 5'CGGCAGCAAGTCTCAGAAACT-3'<br>NBPF19_4: 5'-CGCGCCTCGGAAAGAATAACAG-3'	As above	As above	N/A

RP-PCR Repeat Primed PCR, 7 deaza-dGTP 7-Deaza-2'deoxyguanosine-5'triphosphate, dATP deoxyadenosine triphosphate, dCTP deoxycytodine triphosphate, dTTP deoxythymidine triphosphate, gDNA genomic DNA, $MgCl_2$ *magnesium chloride*

Supplementary Figure 1

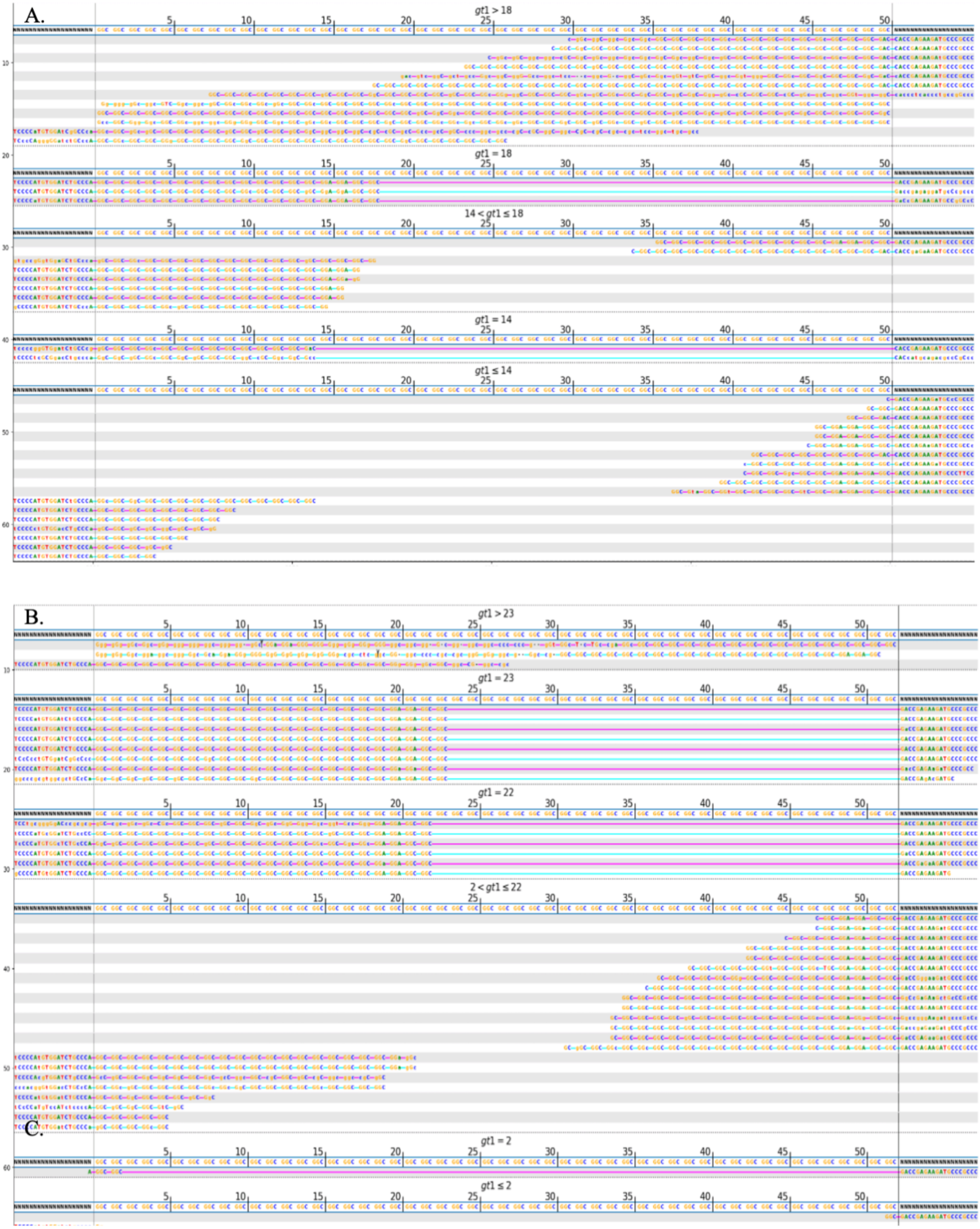

Supplementary Figure 2

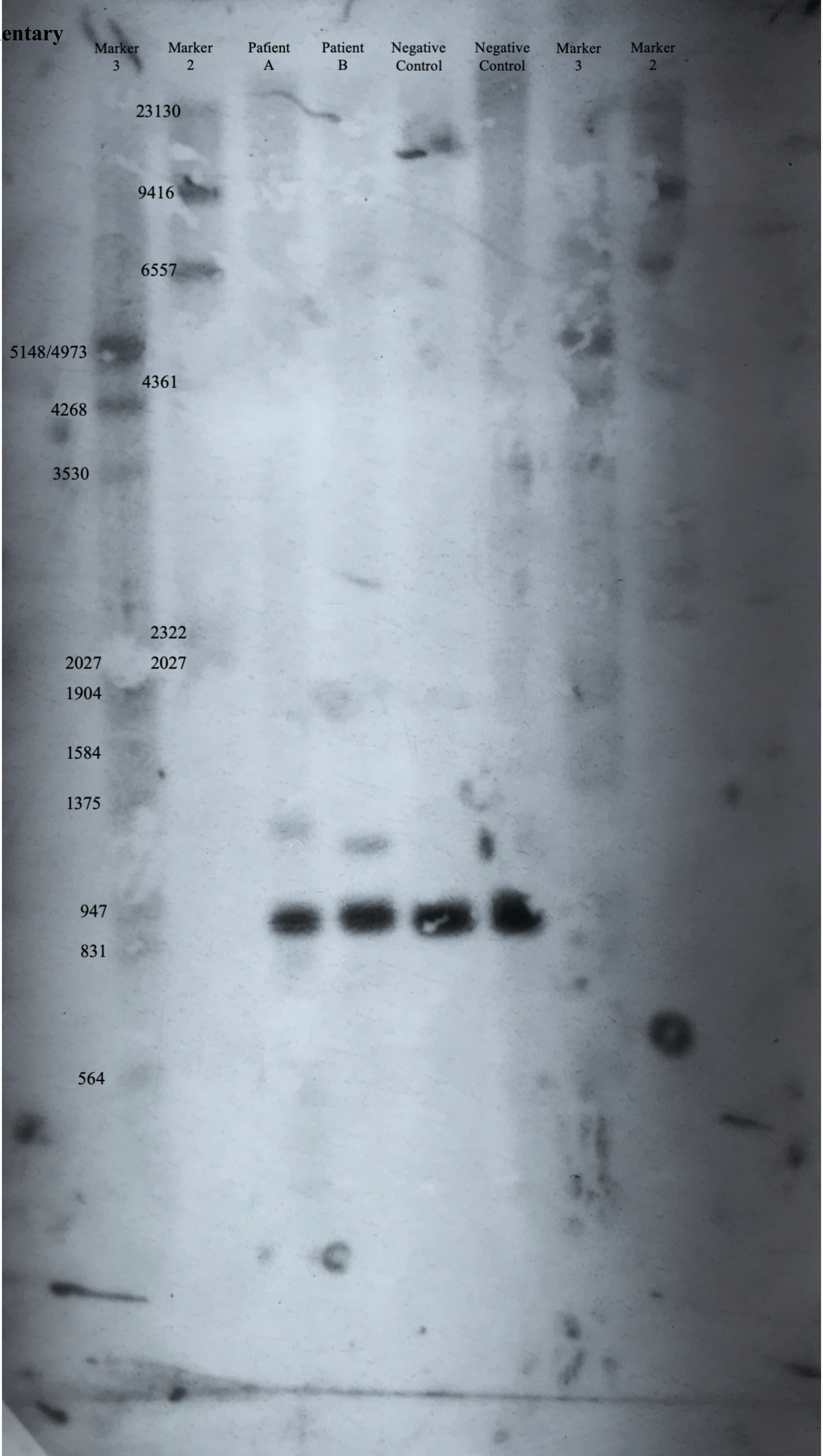

Supplementary Figure 3

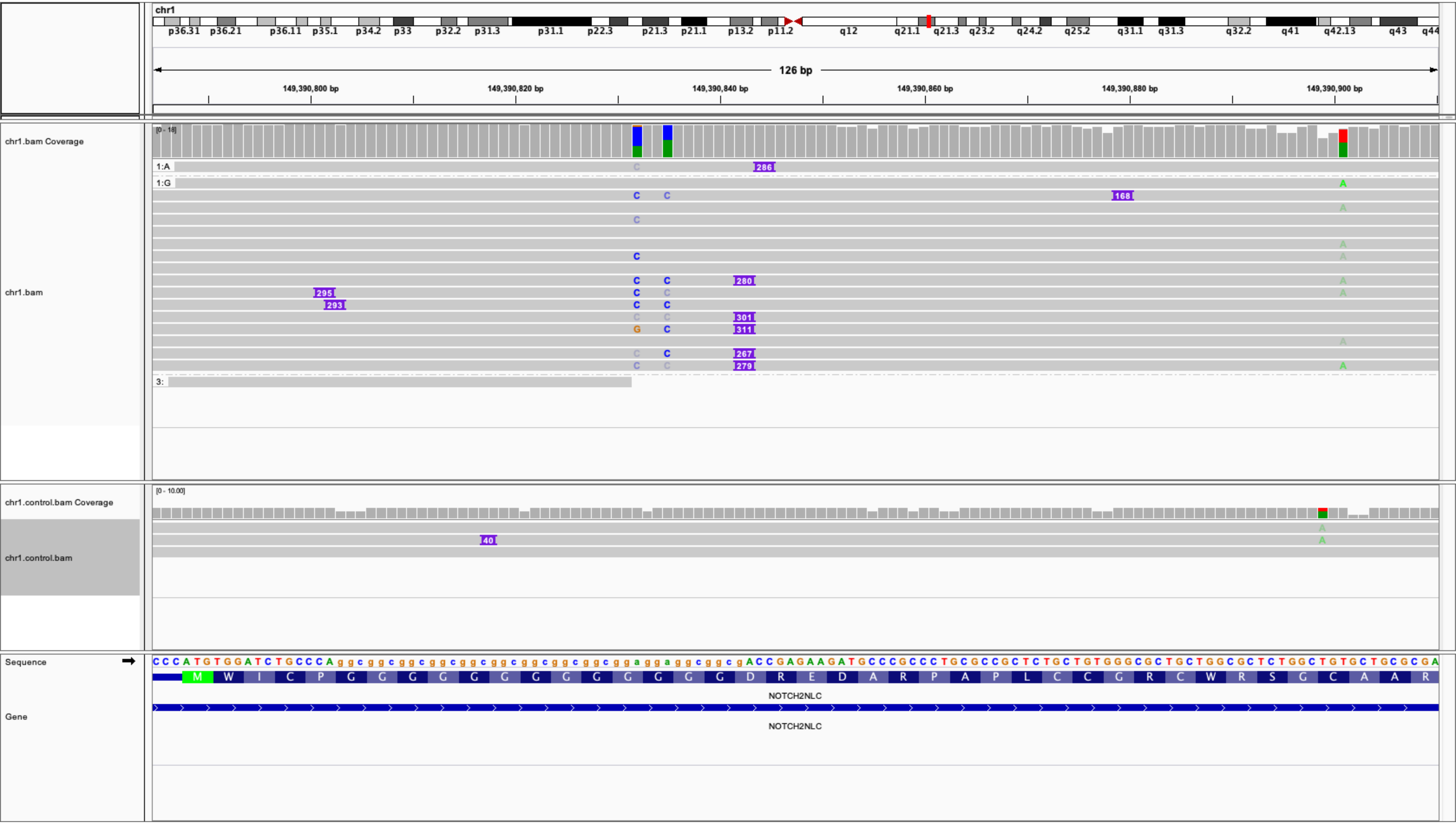

Supplementary Figure 4

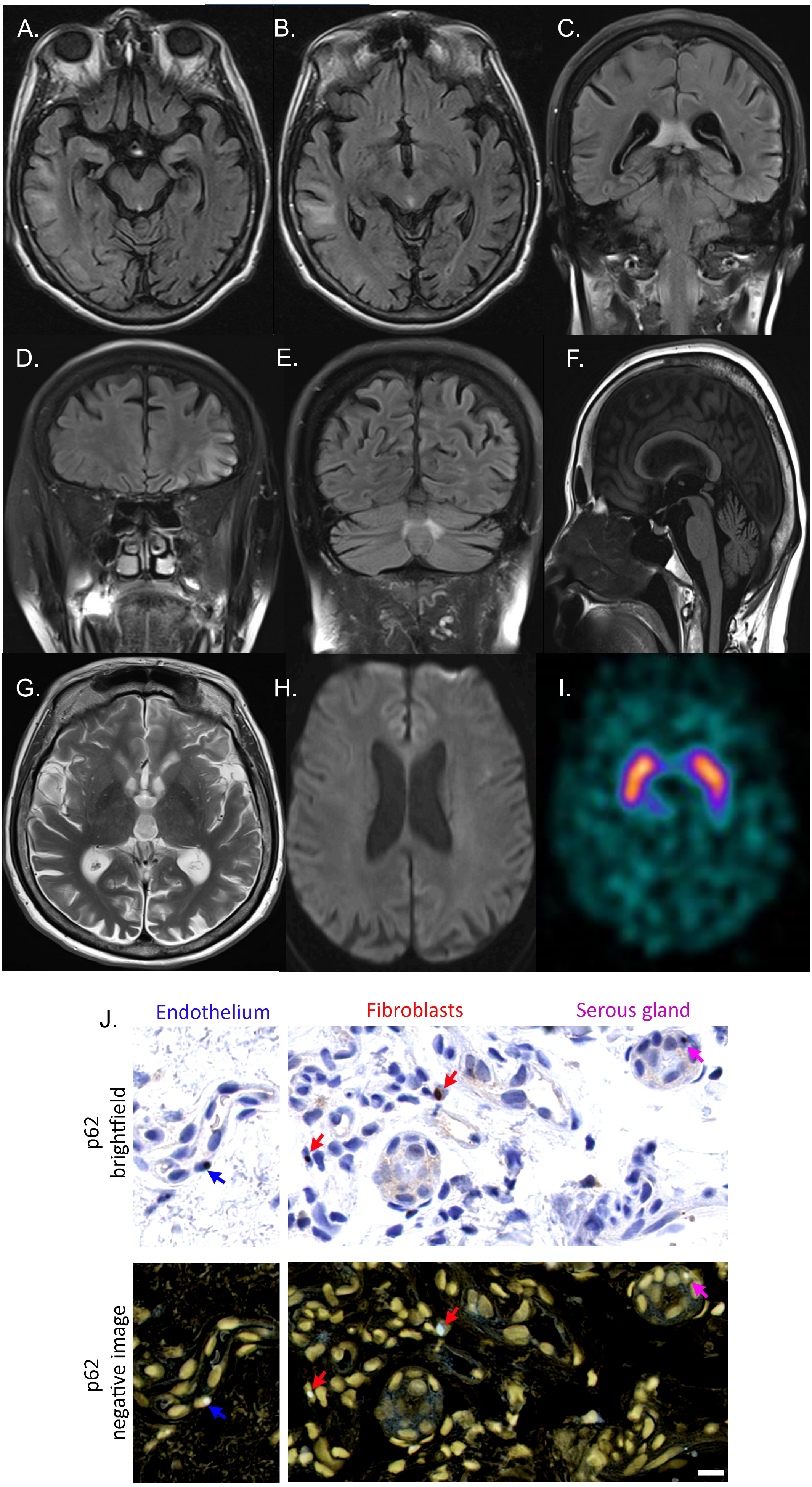

Supplementary figure 5. In-depth case review of patient A.

Patient A is a 60-year-old Ukrainian woman who first developed encephalopathy with migraine and focal neurological symptoms from the age of 39 years. The initial neurological deficits manifested as hemi-visual disturbance, dysphasia, dysarthria and hemisensory loss, lasting for days. Subsequently, she developed chronic migraines with visual/ sensory aura and vertigo. She has continued to suffer from recurrent encephalopathy at least twice a year. At the age of 49 years, she was treated for an episode of possible viral encephalitis when she presented with headache, vomiting, right hemi-visual loss with MRI features of left occipital and temporoparietal grey matter T2-weighted hyperintensity without restricted diffusion. The CSF was bland and sterile except for raised protein 0.61, but viral PCR was not performed. Her symptoms improved over several weeks, but subsequent MRI head showed mild generalised cerebral atrophy. One year later she was treated for breast cancer, which required wide local excision and axillary clearance followed by adjuvant chemo/radiotherapy. PET scan did not identify additional FDG-avid lesions; neuronal antibodies and autoimmune encephalitis antibodies (Anti-GAD, Anti-LG1, Anti-Caspr, anti-glycine and anti-NMDA) were negative. A muscle biopsy showed no pathological evidence of mitochondrial disease and whole mitochondrial sequencing did not detect pathogenic mutation.

Her next major episode of encephalopathy occurred at aged 56 years: five days of worsening headache and vomiting with left hemi-visual loss, followed by focal motor seizures with impaired awareness and secondary generalisation. She was admitted to intensive care unit for treatment of status epilepticus. Her CSF was bland with elevated protein 0.79. Serum ammonia was raised at 58 in the context of multiple antiepileptic drugs, but serum lactate was normal. MRI head on admission showed hyperintensity involving the cortex and the

subcortical white matter of the right temporal and inferior parietal lobes, and splenium of corpus callosum on T2 FLAIR sequences; there was no evidence of vessel abnormality. Patient A recovered over two months, corresponding with improvement in the MRI signal changes. Subsequent video EEG telemetry demonstrated frequent interictal spikes/ waves in the right frontocentral and temporal regions; however, focal neurological symptoms associated with her migraines did not have EEG correlates. Nerve conduction showed axonal sensory neuropathy. Further investigations that yielded normal results include vitamin B12, folate, serum white cell enzymes, amino acids, acylcarnitine profile, pyruvate/ lactate ratio, very long chain fatty acid, urine organic acids, urine porphyrins and porphobilinogen screen. Follow-up MRI head for patient A at age 60 showed a new large area of cortical and subcortical hyperintense signal abnormality involving the left fronto-temporal lobes and middle cerebellar peduncles on T2 FLAIR sequences without hyperintensity in the corticomedullary junction in DWI. She was not encephalopathic and she did not have seizure at the time of the scan.

Her current medications comprise levetiracetam, aspirin, olanzapine, rosuvastatin and omeprazole. She is a non-smoker and a teetotaller. She had normal developmental milestones and attended tertiary education qualification in Ukraine. She is the only person affected in the family.

Neurological examination at age 60 years revealed a dexterous Caucasian woman with mild dysarthria, hypomimia and mild cognitive impairment. She did not have postural blood pressure drop. Her tone was not increased. She had resting and postural tremor with mild bilateral bradykinesia and hypokinesia. Her strength was full and was globally areflexic. Her plantar responses were down-going. She had reduced sensation to pinprick to knees and

vibration sense to ankles, but other modalities were intact. Her gait, postural balance, cranial nerve examinations were normal. There was no evidence of dysmetria or apraxia.

DaT scan was performed subsequent to the genetic diagnosis and did not show reduced uptake of dopamine transporter in the nigrostriatal system. Skin biopsy revealed p62 positive intranuclear inclusions in endothelium, fibroblast and serous glands.

Supplementary Figure 6

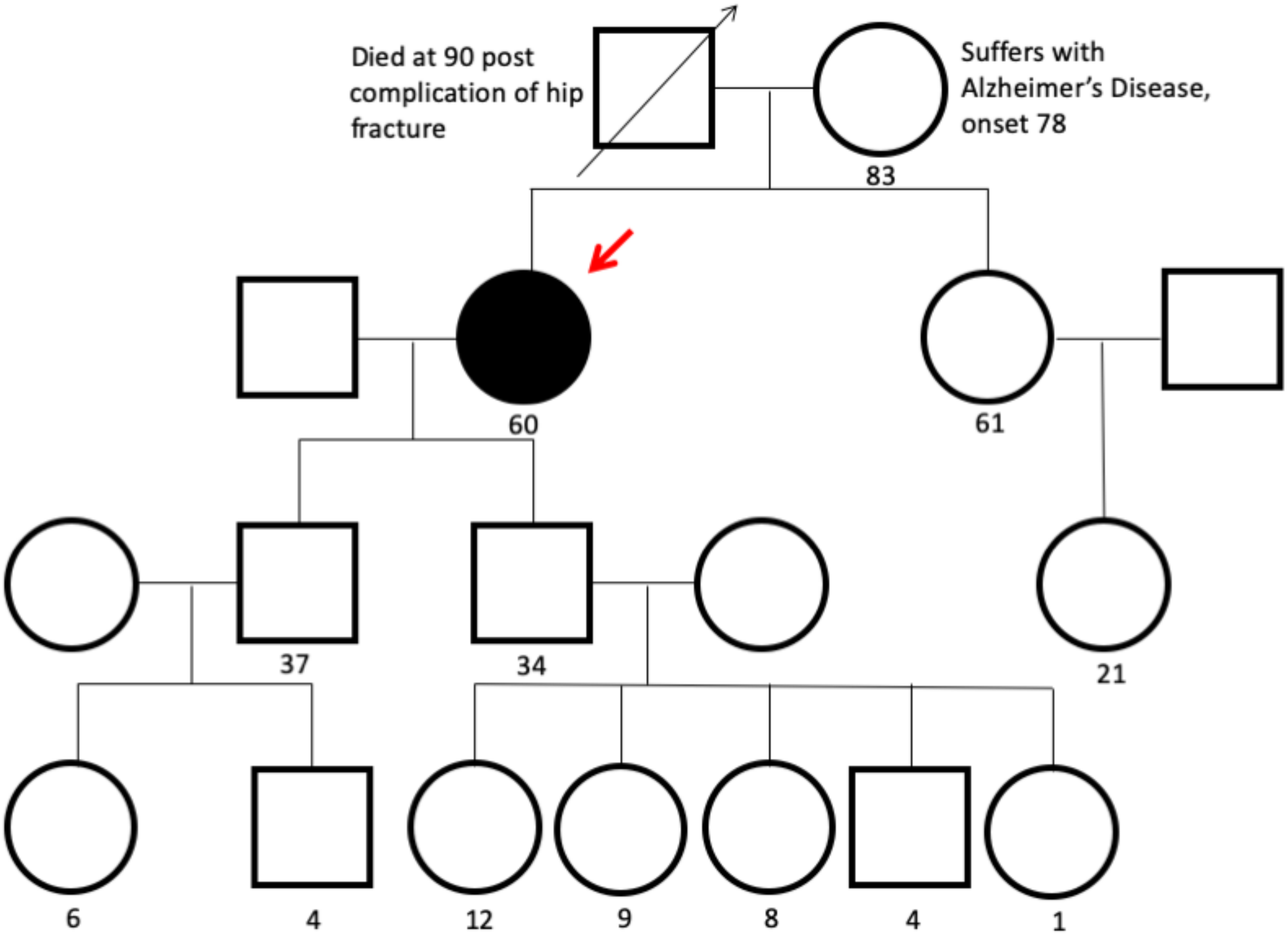

Supplementary Figure 7

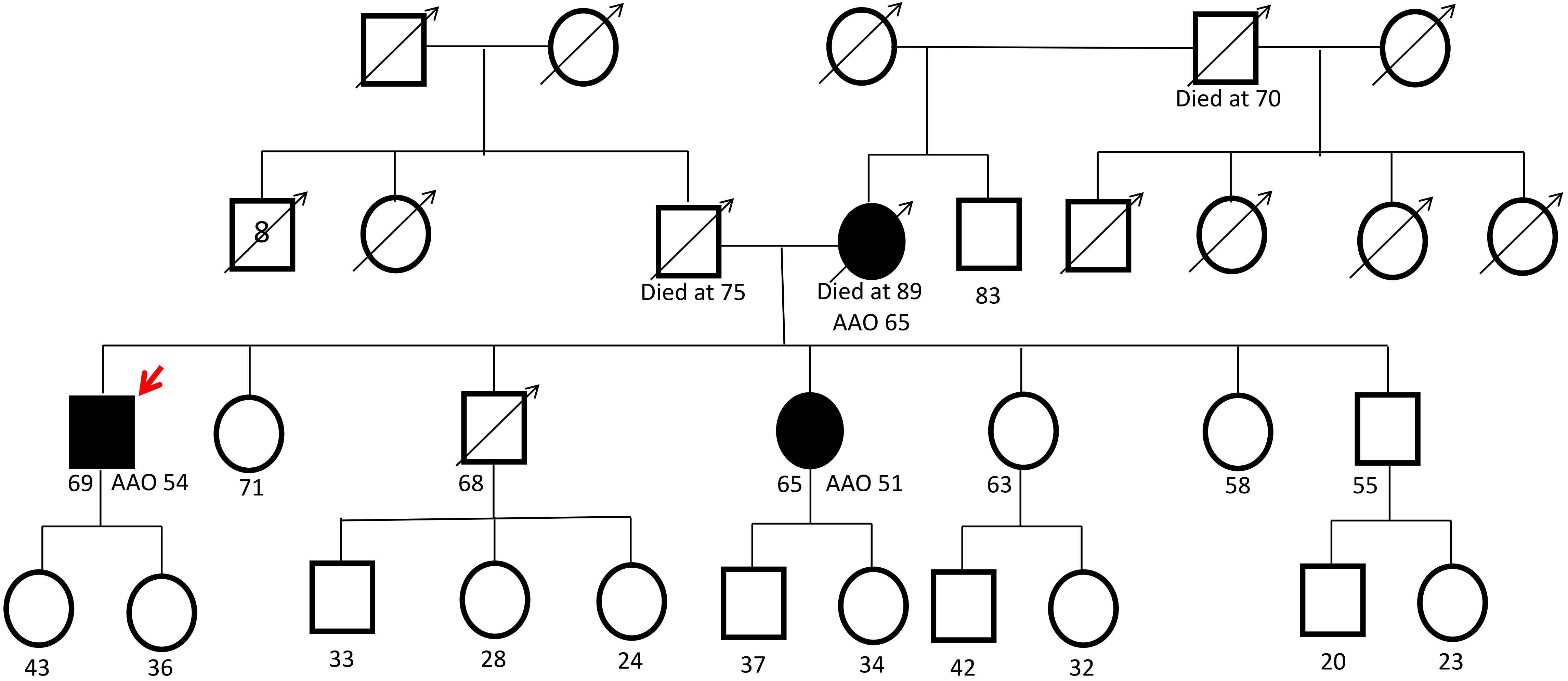

Supplement: Supplementary file 2 — Table S1. Primer sequences and thermocycling conditions Figure S1. A visual representation of ExpansionHunter output generated by GraphAlignmentViewer for the proband captured multiple reads of pure GGC‐repeat tracts of various lengths (A) compared with the output of the false‐positive cases showing interrupted GGC‐repeat tracts and a smaller number of reads supporting the expanded allele (B). Figure S2. Southern blot uncropped image. Figure S3. Integrative Genomics Viewer of Oxford Nanopore Technologies long‐read sequencing uncropped image. Figure S4. MRI head, DaTscan, and skin histopathology images of patient A. MRI head of patient A at age 49 years during an episode of encephalopathy showed hyperintensities in the right temporoparietal cortical and subcortical regions and corpus callosum in T2 FLAIR sequences (A–C); routine scan with patient at age 60 years showed hyperintensities in left frontal gyrus and middle cerebellar peduncles in T2 FLAIR coronal sequences (D–E) and mild global cerebral atrophy in T1 sagittal sequence (F) but no evidence of nigrostriatal degeneration on T2‐weighted axial sequences or hyperintensity in the corticomedullary junction in DWI sequences (G–H). DaTscan showed normal uptake of striatal dopamine transporter (I). Skin biopsy of patient A showed p62‐positive intranuclear inclusions in endothelium (blue arrow), fibroblasts (red arrow), and serous glands (pink arrow). Upper row demonstrates p62 immunostaining with hematoxylin counterstain when viewed under a bright‐field microscope, and the bottom row shows the negatives of the bright‐field images. Scale bar: 10 μm (J). Figure S5. In‐depth case review of patient A. Figure S6. Four‐generation family tree of patient A. The number below an individual indicates the age in years. Figure S7. Four‐generation family tree of patient B. The number below ane individual indicates the age in years. [file MDS-36-251-s003.pdf]
