# Supplementary material for: Low Prevalence of NOTCH2NLC GGC Repeat Expansion in White Patients with Movement Disorders
Source: Mov Disord. 2020 Oct 7;36(1):251–5. doi: 10.1002/mds.28302 (PMC8436747; doi:10.1002/mds.28302)
Supplement: Supplementary file 3 — Appendix S1. Members of Genomics England Research Consortium [file MDS-36-251-s001.docx]

**Appendix 1. Members of Genomics England Research Consortium**

Ambrose J. C. ^1^ , Arumugam P.^1^ [
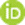
](https://orcid.org/0000-0002-2444-0698), Baple E. L. ^1^ [
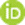
](https://orcid.org/0000-0002-6637-3411), Bleda M. ^1^ [
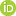
](https://orcid.org/0000-0002-1287-6013), Boardman-Pretty F. ^1,2^ [
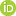
](https://orcid.org/0000-0001-8459-4739), Boissiere J. M. ^1^ , Boustred C. R. ^1^ , Brittain H.^1^ [
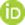
](https://orcid.org/0000-0002-1582-0031), Caulfield M. J.^1,2^ , Chan G. C. ^1^ , Craig C. E. H. ^1^ [
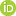
](https://orcid.org/0000-0002-1150-5501), Daugherty L. C. ^1^ [
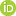
](https://orcid.org/0000-0003-4546-6667), de Burca A. ^1^ , Devereau, A. ^1^ , Elgar G. ^1,2^ [
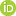
](https://orcid.org/0000-0001-7323-1596), Foulger R. E. ^1^ [
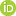
](https://orcid.org/0000-0001-8682-8754) , Fowler T. ^1^ [
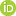
](https://orcid.org/0000-0002-7258-2279), Furió-Tarí P. ^1^ [
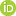
](https://orcid.org/0000-0002-2327-6830), Hackett J. M. ^1^ , Halai D. ^1^ , Hamblin A.^1^, Henderson S.^1,2^, Holman J. E. ^1^ , Hubbard T. J. P. ^1^ [
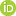
](https://orcid.org/0000-0002-1767-9318), Ibáñez K.^1,2^ [
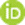
](https://eur01.safelinks.protection.outlook.com/?url=https%3A%2F%2Forcid.org%2F0000-0002-7718-814X&data=02%7C01%7Csamuel.smith%40genomicsengland.co.uk%7Cb1f3c59abe024722a16408d7400c070c%7C569df091b01340e386eebd9cb9e25814%7C0%7C0%7C637048292725351433&sdata=kEg9gQhKO%2BqR0JuAWT%2FRvv5ILABK9h14V39rkLADD5M%3D&reserved=0), Jackson R. ^1^ [
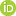
](https://orcid.org/0000-0001-6987-5954), Jones L. J. ^1,2^, Kasperaviciute D. ^1,2^ , Kayikci M. ^1^ [
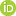
](https://orcid.org/0000-0002-0170-1939), Lahnstein L. ^1^ , Lawson K. ^1^ [
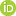
](https://orcid.org/0000-0001-8024-8177), Leigh S. E. A. ^1^ [
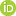
](https://orcid.org/0000-0003-3368-7059), Leong I. U. S. ^1^ [
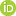
](https://orcid.org/0000-0002-0831-3461), Lopez F. J. ^1^ , Maleady-Crowe F. ^1^ , Mason J. ^1^ [
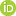
](https://orcid.org/0000-0001-6700-5971), McDonagh E. M. ^1,2^  [
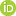
](https://orcid.org/0000-0001-5806-6174), Moutsianas L. ^1,2^  [
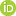
](https://orcid.org/0000-0001-5453-345X), Mueller M. ^1,2^ [
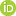
](https://orcid.org/0000-0002-2056-1185) , Murugaesu N. ^1^, Need A. C. ^1,2^ [
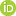
](http://orcid.org/0000-0003-3955-8207), Odhams C. A. ^1^ [
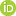
](https://orcid.org/0000-0003-2396-6150), Patch C. ^1,2^ [
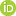
](https://orcid.org/0000-0002-4191-0663), Perez-Gil D. ^1^ , Polychronopoulos D. ^1^ [
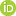
](https://orcid.org/0000-0001-9427-3082), Pullinger J. ^1^ [
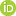
](https://orcid.org/0000-0001-9357-1184), Rahim T. ^1^ [
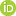
](https://orcid.org/0000-0001-6504-0618), Rendon A. ^1^ [
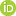
](https://orcid.org/0000-0001-8994-0039), Riesgo-Ferreiro P.^1^ [
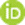
](https://orcid.org/0000-0003-2739-1315), Rogers T. ^1^ , Ryten M. ^1^ , Savage K. ^1^ , Sawant K. ^1^, Scott R. H. ^1^ , Siddiq A. ^1^ [
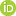
](https://orcid.org/0000-0002-9110-1434), Sieghart A. ^1^ [
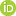
](https://orcid.org/0000-0001-5484-4346), Smedley D. ^1,2^ , Smith K. R. ^1,2^ [
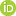
](https://orcid.org/0000-0002-0329-5938), Sosinsky A. ^1,2^ [
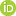
](https://orcid.org/0000-0001-9022-7409), Spooner W. ^1^ [
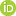
](https://orcid.org/0000-0001-9226-5919), Stevens H. E. ^1^ [
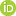
](https://orcid.org/0000-0003-0836-5875), Stuckey A. ^1^ [
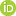
](https://orcid.org/0000-0001-8636-737X), Sultana R. ^1^, Thomas E. R. A. ^1,2^ [
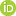
](https://orcid.org/0000-0001-7412-1962), Thompson S. R. ^1^ [
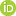
](https://orcid.org/0000-0001-6966-5842), Tregidgo C. ^1^ , Tucci A. ^1,2^ [
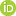
](https://orcid.org/0000-0001-5644-0070), Walsh E. ^1^ [
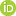
](https://orcid.org/0000-0001-5094-2723), Watters, S. A. ^1^ [
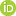
](https://orcid.org/0000-0001-6628-1261), Welland M. J. ^1^ , Williams E. ^1^ [
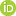
](https://orcid.org/0000-0002-0851-7990), Witkowska K. ^1,2^ , Wood S. M. ^1,2^, Zarowiecki M.^1^ [
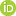
](https://orcid.org/0000-0001-6102-7731).

1. Genomics England, London, UK

2. William Harvey Research Institute, Queen Mary University of London, London, EC1M 6BQ, UK.
